# 

**Published:** 1890-10

**Authors:** 


### CONTENTS:

Article I.—On the Dangei \rising from Syphilis in the Practice of Dentistry. By L. Duncan Bulkley. - 241

" II.—The Hypnotism Craze and its Dangers. - - - 250

" III.—The Odontoblasts : Their Formation, Function and Power of Appropriation. - - - - - 252

" IV.—The Chemistry of Dental Caries. By R. C. Young, D.D.S. - - - - - - 260

" V.—Some Historic Points and Practical Hints on Crowning. By Thos. G. Read, L.D.S. - - 265

" VI.—Current Dental Literature. By Geo. W. Williams. - - - - - - 273

" VII.—Dangers from Necrosed Teeth. - - - 275

" VIII.—How to Deal with another Man's Patients. - - 277

" IX.—An Anomalous Case on Salivary Calculus. By Edmund Owen, F.R.C.S. - - - - 279

### EDITORIAL.

Cholera Precautions Suggested. - - - - - - 282

Washington's Tooth. - - - - - - - - - 282

Getting Ahead of St. Paul. - - - - - - - 283

Trade in Cast-off Teeth. - - - - - - - 284

### MONTHLY SUMMARY.

Dangers of Hypnotism.. - - - - - - - 285

Danger in Exercise. - - - - - - - - 286

Pyorrhea Alveolaris Complicated with Necrosis. - - 286

Hypodermic Injections of Ergot in Facial Neuralgia. - - 287

A Practical method of Electro-Gilding Gold Dentures, &c. - 288

